# GRAPE: A multi-modal dataset of longitudinal follow-up visual field and fundus images for glaucoma management

**DOI:** 10.1038/s41597-023-02424-4

**Published:** 2023-08-05

**Authors:** Xiaoling Huang, Xiangyin Kong, Ziyan Shen, Jing Ouyang, Yunxiang Li, Kai Jin, Juan Ye

**Affiliations:** 1 Eye Center, The Second Affiliated Hospital, School of Medicine, Zhejiang University, Hangzhou, 310003 China; 2 State Key Laboratory of Industrial Control Technology, College of Control Science and Engineering, Zhejiang University, Hangzhou, 310013 China; 3 Zhejiang Baima Lake Laboratory Co., Ltd, Hangzhou, 310051 China; 4 Department of Radiation Oncology, UT Southwestern Medical Center, Dallas, TX 75235 USA

**Keywords:** Optic nerve diseases, Scientific data

## Abstract

As one of the leading causes of irreversible blindness worldwide, glaucoma is characterized by structural damage and functional loss. Glaucoma patients often have a long follow-up and prognosis prediction is an important part in treatment. However, existing public glaucoma datasets are almost cross-sectional, concentrating on segmentation on optic disc (OD) and glaucoma diagnosis. With the development of artificial intelligence (AI), the deep learning model can already provide accurate prediction of future visual field (VF) and its progression with the support of longitudinal datasets. Here, we proposed a public longitudinal glaucoma real-world appraisal progression ensemble (GRAPE) dataset. The GRAPE dataset contains 1115 follow-up records from 263 eyes, with VFs, fundus images, OCT measurements and clinical information, and OD segmentation and VF progression are annotated. Two baseline models demonstrated the feasibility in prediction of VF and its progression. This dataset will advance AI research in glaucoma management.

## Background & Summary

Glaucoma is a degenerative optic neuropathy characterized by the loss of retinal ganglion cells that frequently causes irreversible blindness^1,2^. It has affected more than 64.3 million individuals worldwide and the number may be increased to 111.8 million in 2040^3^. It has different subtypes and a wide range of inconspicuous risk factors, such as the optic nerve head (ONH) damage, visual field (VF) loss and intraocular pressure (IOP) elevation^4^. Timely detection and treatment can prevent glaucoma from getting worse^5^.

**Fig. 1:**
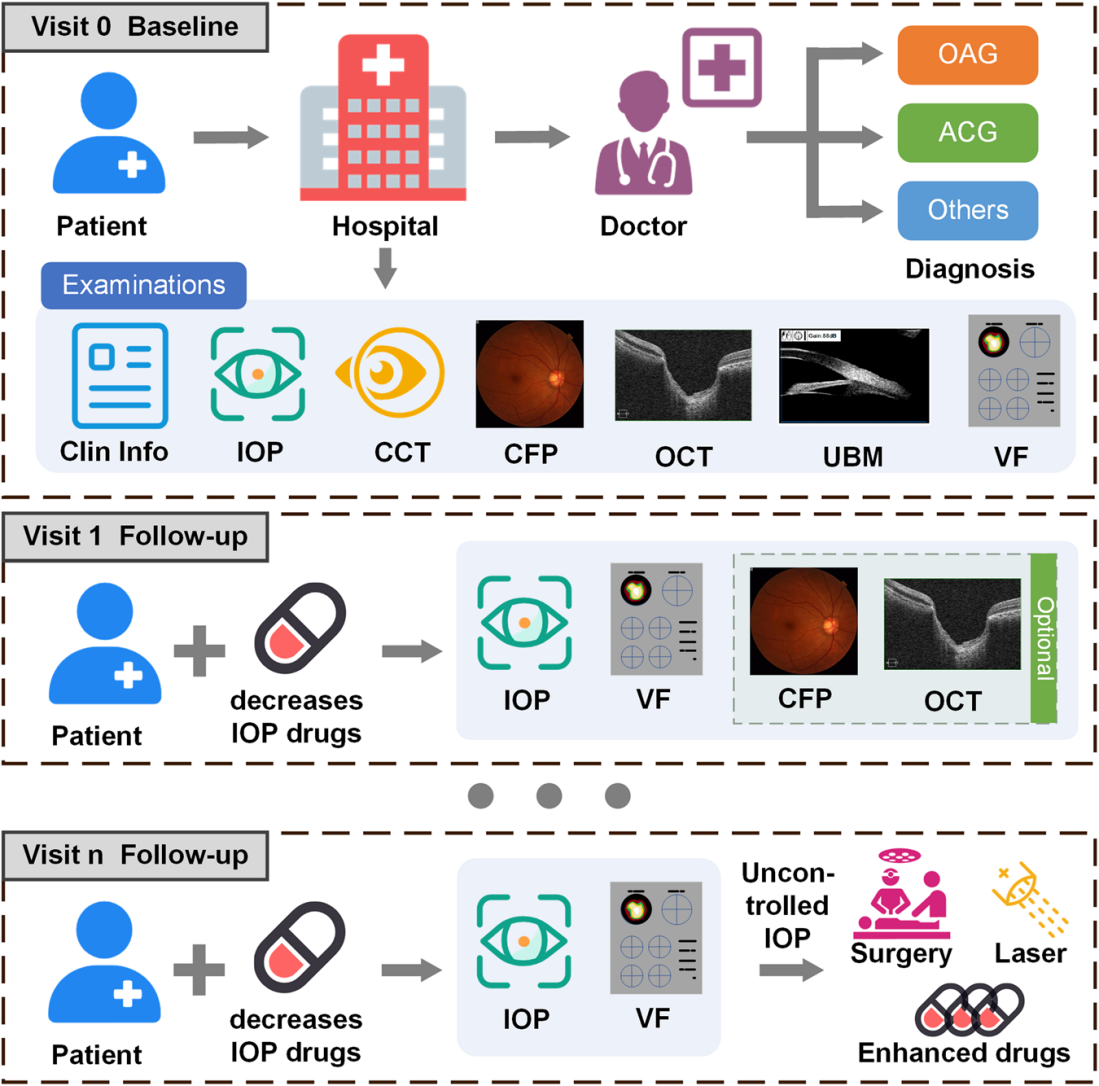
The routine medical process of glaucoma patients. The complete examinations tend to be accomplished to confirm the diagnosis at the first time of one patient. Subsequently, the patient with decreasing IOP drugs would do regular follow-up to check IOP, VF and other examinations. If IOP is uncontrolled, additional medical treatments, laser treatments and surgical treatments are necessary.

Due to the difficulty in treatment, the glaucoma patients are often accompanied with a chronic progressive course^6^. Regular follow-ups with VF reviewing by standard automated perimetry (SAP) and IOP monitoring show great significance to the visual function evaluation of glaucoma patients^7^. The color fundus photograph (CFP) and optical coherence tomography (OCT) are implemented to detect structural damage. The routine examinations during follow-up of one glaucoma patient are shown in Fig. 1. At the first time, relatively complete examinations would be applied of one suspected glaucoma patient, for the doctor to generate the preliminary diagnosis. Then the patient with intraocular hypertension would take the medicine to decrease IOP to prevent further deterioration of visual function, and visit regularly to check IOP and VF, sometimes fundus structure if necessary. If IOP is uncontrolled, additional medical treatments, laser treatments and surgical treatments would be performed.

With the development of artificial intelligence (AI), several researches have payed attention to glaucoma management, and used several biometric parameters or medical images to predict the onset and progression of glaucoma^8–12^. On account of the considerable test-retest variability and strong patient cooperation requirement of VF tests^13–15^, previous studies have also proposed various machine learning (ML) and deep learning (DL) models to predict VF from structural parameters or images, with tolerable estimation error^16–19^. The development of these AI models about glaucoma assessment needs complete follow-up examination data of patients.

Many well-known glaucoma public datasets of retinal images have been established for automated glaucoma screening, including RIGA^20^, ORIGA^21^, DRISHTI-GS^22^, REFUGE^23^ and PAPILA^24^. These datasets mainly concentrated on the segmentation of optic disc (OD) and optic cup (OC), emphasizing the discrepancy on fundus characteristics between glaucoma and normal patients. However, there are few public datasets about glaucoma progression research, and some are listed below:

- UWHVF^25^: This is an open-source VF dataset from the University of Washington with 28943 VFs. It contains longitudinal VFs from one eye or both eyes of one patient. Pointwise sensitivities were extracted from Humphrey Field Analyzer (HFA) with other related VF parameters calculated from it. The progression analysis was performed on part of VFs.
- Annotated Glaucoma Medication Dataset^26^: This is an open-source dataset including 480 clinical notes from the Electronic Health Record (EHR) system. All progress notes were annotated for glaucoma medication name, route, frequency, dosage, and drug use. It is mainly used for natural language processing model development.

In general, most datasets containing CFP or OCT images focused on glaucoma diagnosis, that longitudinal changes generated during follow-up cannot be evaluated. While the datasets for progression detection only include VF values or medical records, without comprehensive reflecting the condition of glaucoma patients. Future VF progression prediction from IOP^27^ or CFP^11^ cannot be realized on these datasets.

In this case, we proposed a glaucoma real-world appraisal progression ensemble (GRAPE) dataset, consisting of the clinical data, VF values, OCT measurements and CFPs at the baseline and during the follow-up visits. It records the full follow-up procedure of glaucoma patients. This dataset could be used for progress prediction and VF estimation of glaucoma. It will promote AI-based researches in glaucoma management.

## Methods

### Patient inclusion

All data in the GRAPE dataset was collected in the Eye Center at the Second Affiliated Hospital of Zhejiang University (ZJU). that contains 1115 records of 263 eyes from 144 glaucoma patients from 2015 to 2022, with ages ranging from 18 to 81 years. We excluded the patients under 18 years old. Written informed consent complying with the requirement of the Medical Ethics Committee of ZJU was signed by every participant. This study is a registered clinical study (A New Technique for Retinal Disease Treatment, ClinicalTrials.gov identifier: NCT04718532). Ethical approval for the study was obtained from Ethics Committee of ZJU-2 (No Y2020–1027). The research adhered to the tenets of the Declaration of Helsinki and the Health Portability and Accessibility Act.

This study only included patients with a definite diagnosis of both open-angle glaucoma (OAG) and angle-closure glaucoma (ACG). The specialists comprehensively considered the glaucoma examination of patients, including medical history, function assessment (VF), structural assessment (CFP or OCT or both), IOP measurement, CCT measurement, anterior chamber angle evaluation. A suspected glaucoma patient would have further visits to confirm the diagnosis. These glaucoma patients were manly diagnosed by senior outpatient clinicians, and subsequently confirmed by 3 glaucoma specialists to be included in this dataset, according to the American Academy of Ophthalmology’s Preferred Practice Pattern of glaucoma guidelines^28,29^. The exclusion criteria are: (1) patients with other optic nerve diseases; (2) patients with severe retinal diseases (e.g. vitreous hemorrhage and retinal detachment); (3) patients with severe dioptric media turbidity; (4) patients who had undergone glaucoma surgery.

### Data collection

The collection process of different data categories is listed below:

#### VF

**Fig. 2:**
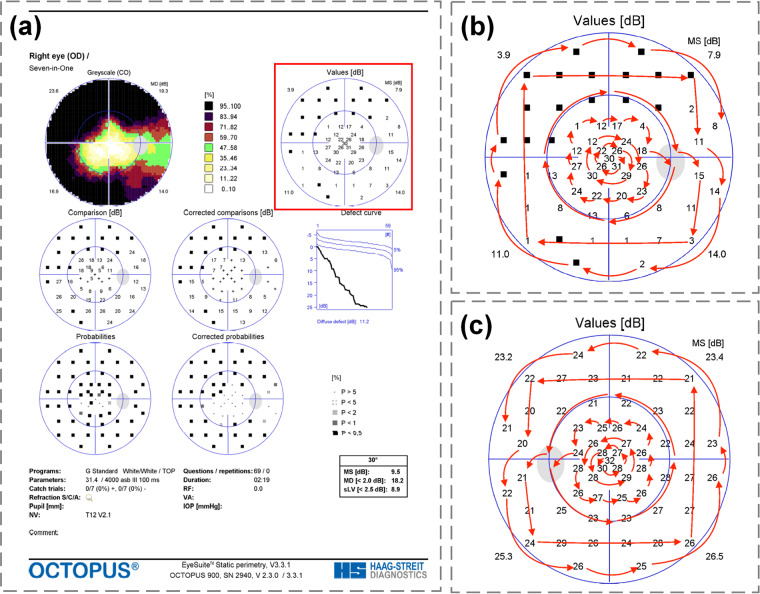
The visual field (VF) report and the extraction order of the values. (**a**) an example of a VF report from Octopus perimeter. We used the light sensitivity values in the “Values” in the red box in the GRAPE dataset. (**b**) The extraction order of VF values of the right eye. (**c**) The extraction order of VF values of the left eye.

The VFs included in the GRAPE dataset were measured by two experienced technicians using the G1 program test pattern with stimulus size III by OCTOPUS 900 perimeter (HAAG-STREIT, Switzerland). The VFs with false-negative rate ≥ 30% or false-positive rate ≥ 30% were identified as unreliable examinations and were excluded^30,31^. The VF values were extracted from the “Values” on VF reports, as shown in Fig. 2a. The “Values” in Octopus VF reports is equal to the light sensitivity values in VF reports measured by Humphrey Field Analyzer (HFA). The VFs were extracted with certain order that is mirror for left and right eyes to ensure all the VFs with the same format, as right eye shown in Fig. 2b and left eye shown in Fig. 2c. The black square, “■”, indicates that this eye is not able to perceive the lowest intensity of light at this location, and we assigned value −1 for it in the dataset. Likewise, two blind points were assigned value “−1”. The VFs in our dataset were finally presented as digital format in the table sheet.

#### CFP

The CFPs were obtained by two experienced technicians using a 50° field of view centered at macula by the TRC-NW8 Fundus Camera (TOPCON, Japan), CR-2 PLUS AF Digital Retinal Camera (CANON, Japan) and CR-2 AF Digital Retinal Camera (CANON, Japan) without pupil dilation. The pictures were saved in JPG format with 3 resolutions of 1556 × 1556 pixels, 2136 × 2136 pixels, and 1611 × 1611 pixels for these three cameras, respectively. We excluded the poor-quality images that 50% of the area is obscured or only part of OD is visible. And we included the image with the best quality if there were more than one image of each eye at the same visit. The image preprocessing of extracting the region of interest (ROI) and annotating the OD segmentation was described below.

#### Retinal nerve fiber layer (RNFL) thickness

The RNFL thickness measurements at baseline in the GRAPE dataset were acquired by two experienced technicians using CIRRUS HD-OCT 5000 (Carl Zeiss Meditec, United States of America). The ONH and RNFL Analysis pattern for Optic Disc 200 × 200 was used to detect and calculate the total and 4 sectoral (superior, nasal, inferior and temporal) mean peripapillary RNFL thickness.

#### IOP

The values of IOP were measured by two technicians using non-contact tonometer NT-530P (NIDEK, Japan). The patients were informed that there would be air ejection during the measurement in advance, to avoid the error caused by the scare for the sudden air ejection. Each eye was automatically measured for 3 times and the average value was used. The glaucoma patients included used the IOP control medications in the follow-up period.

#### Central corneal thickness (CCT)

The values of CCT were measured by two technicians using non-contact tonometer NT-530P (NIDEK, Japan), same to the IOP. The patients were with the correct sitting position and focused on the indicator light, then technicians could measure the value of CCT.

#### Basic clinical data

These clinical data was acquired from the EHR system. The basic data and medical history were inquired by clinicians or nurses during the attendance process.

### Data summary

The data provided in the GRAPE dataset and the format are listed in Table 1. There are two sheets in the table for the baseline data and the follow-up data, respectively. The baseline visit is defined as the first time the patient received a comprehensive evaluation of glaucoma, including medical history, function assessment (VF), structural assessment (CFP, OCT or both), IOP measurement, CCT measurement, anterior chamber angle evaluation. If the patient had prior visit record for other ocular diseases or only with VF examination, this visit record would not be included in the dataset.

**Table 1:** Summarization and detail description of the GRAPE dataset.

	Data	Format	Detail description
Baseline	Age	Number	The age at the first visit
	Gender	F/M	“F” for female and “M” for male
	CCT	Number	The value of CCT
	Times of visits	Number	Times of visits during follow-up
	Progression-PLR2	0/1	“0” for no progression, and “1” for progression
	Progression-PLR3	0/1	“0” for no progression, and “1” for progression
	Progression-MD	0/1	“0” for no progression, and “1” for progression
	Category	OAG/ACG	The two categories of glaucoma
	OCT RNFL thickness	A group of number	Including mean (“Mean”), superior (“S”), nasal (“N”), inferior (“I”) and temporal (“T”) peripapillary RNFL thickness
Baseline & follow-up	Subject number	Number	The serial number of inclusion subjects in this dataset
	Laterality	OD/OS	The laterality of one eye, “OD” for right eye and “OS” for left eye
	Visit number	Number	The times for follow-up visits from baseline
	Interval year	Number	The interval years between each visit and baseline
	IOP	Number	The value of IOP
	VF	A group of number	Including the Octopus VF values of 61 points
	CFP	Image	Including the orginal CFP, ROI and ROI with OD/OC annotation
	Acquisition Device	Device name	The devices taking the CFPs, including 3 types of devices
	Resolution	Number × number	The resolution of CFP, corresponding to the Acquisition Device, including 3 types, 1556 × 1556, 2136 × 2136, 1611 × 1611

There are 263 multi-modal records in the baseline data, including subject number, laterality, age, gender, IOP, CCT, total visits, progression status, category of glaucoma, OCT RNFL thickness, corresponding CFP, acquisition device, resolution and VF. And there are 1115 visit records in follow-up data, consisting of subject number, laterality, visit number, interval years, IOP, corresponding CFP, acquisition device, resolution and VF. The quantification methods of progression are introduced below. Besides, the original CFPs were placed in one folder, while the processed images and the annotation files were placed in another folder. The image preprocessing method is also introduced below.

### Image preprocessing

**Fig. 3:**
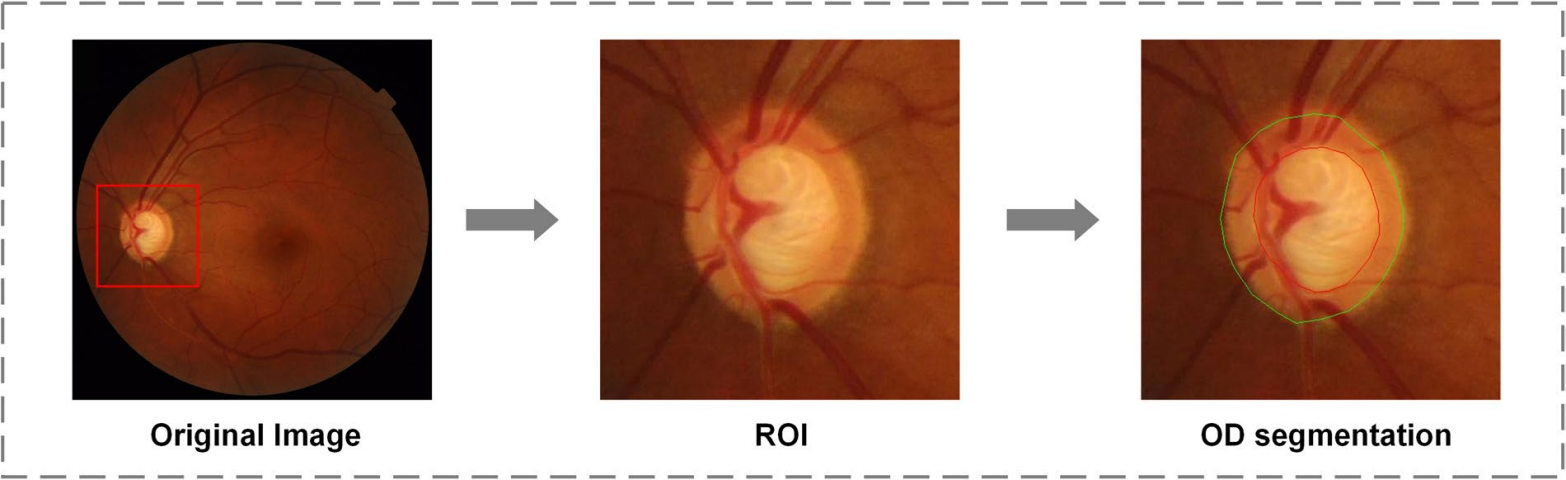
The preprocessing procedure of color fundus photographs (CFPs). The region of interest (ROI) was clipped from the original image, and the annotation of optic disc (green line) and optic cup (red line) was labeled by experienced ophthalmologist on ROI.

The fundus manifestation of glaucoma contains OD rim narrowing, cup-to-disc ratio (CDR) increasing, large extent of parapapillary atrophy, and RNFL defect^28,29^, mainly focused on the area around OD. For AI-based model to identify the characteristics more facilely, ROI, a square area around OD, was clipped by one experienced ophthalmologist. Generally, the OD was regarded as the center of the clipped image. Besides, in view of the large extent of parapapillary atrophy is one of the major features, we preserved this structure in the ROI as possible. The ROIs of CFPs were cropped into 453 × 453 pixels, 566 × 566 pixels and 432 × 432 pixels for three devices, respectively. The whole procedure of image disposing is shown in Fig. 3. In addition, we hid all personal information on CFPs.

To singularize the feature of CDR increasing and OD rim narrowing, OD and OC segmentation on ROI images was performed by one ophthalmologist using the label tool. The red circle is the border of OC and green of OD in the annotated images. We also provided the original annotation files, JSON files, that record the XY coordinates, and the codes that could draw the label line on the ROI images.

### Quantification of VF progression

The Comparison Values, that are the total deviation (TD) values on HFA reports, were calculated by light sensitivity values and normative values. The normative values were obtained from the device according to the different ages. TD values were calculated for all 59 locations excluding the two blind spots, with the locations same to that of light sensitivity values. Because the TD values less than 5 were displayed as “ + ” in Comparison of Octopus VFs, we calculated the TD values according to the original sensitivity values and age, conforming to the definition provided by the Imaging and Perimetry Society^32^.

There are several different methods for detecting glaucomatous VF progression^33,34^. We chose three commonly used automated progression criteria, two based on point-wise linear regression (PLR) analysis and one based on mean deviation (MD) slope, on account of the fitness for Octopus VFs. For PLR, the baseline VF of one eye is defined as progressing if the number of test points with a significant negative regression slope (P < 0.01) greater than or equal to two (PLR2) or three (PLR3). For MD slope, when the slope of MD over follow-up time is presented with negative linear regression and P value is less than 0.05, this eye is regarded as progression. We calculated the progression by the visual Felds package^35^, an open-source software to analyze VF based on R environment. This package has been modified to be compatible with Octopus VF.

## Data Records

The GRAPE dataset is available at Figshare^36^. The main part of the dataset includes 6 parts, 1 Excel file, 4 zipped files and 1 python file. The zipped file could be decompressed to the corresponding folders.

The Excel file, “VF and clinical information”, contains 2 sheets, titled “Baseline” and “Follow-up”. There are 263 records representing 263 eyes and 80 columns in sheet “Baseline”, the last 61 columns of which are “VF”. These columns are in turn: “Subject Number” (inclusion subjects numbered as 1–144), “Laterality” (OD for right eye and OS for left eye), “Age”, “Gender” (M for male and F for female), “IOP”, “CCT”, “Total Visits” (the total times of visits for each eye), “Progression Status-PLR2” (progression status defined by PLR2 method, 0 for non-progression and 1 for progression), “Progression Status-PLR3” (progression status defined by PLR3 method, 0 for non-progression and 1 for progression), “Progression Status- MD” (progression status defined by MD method, 0 for non-progression and 1 for progression), “Category of Glaucoma” (OAG for open-angle glaucoma and ACG for angle-closure glaucoma), “OCT RNFL thickness” (“Mean” for total mean RNFL peripapillary thickness, “S” for superior, “N” for nasal, “I” for inferior, and “T” for temporal), “Corresponding CFP” (named as “subject number_laterality_visit number. jpg”), “Acquisition Device” (the device that taking the corresponding CFP), “Resolution” (the resolution of the corresponding CFP) and “VF” (the last 61 columns). Besides, there are 1115 records representing 1115 visits and 69 columns in sheet “Follow-up”, the last 61 columns of which are “VF” as well. These columns are in turn: “Subject Number”, “Laterality”, “Visit Number” (the times for follow-up visit from baseline), “Interval Years” (the interval years for follow-up visit from baseline), “IOP”, “Corresponding CFP”, “Acquisition Device”, “Resolution” and “VF”. The symbol “/” in the sheet represents that no corresponding CFP or OCT examination was generated in this visit. The detailed description of the dataset is shown in Table 1.

The folder “CFPs” contains 631 original CFPs from non-mydriatic digital camera, with personal information hidden. These images are named as “subject number_laterality_visit number. jpg”, corresponding to the records in Excel file.

The folder “ROI images” contains 631 ROI of CFPs cropped from the original images, and the naming of them is consistent with that of original images.

The folder “Annotated images” includes 631 ROI images with OD/OC segmentation. The green contour is for OD and red for OC, respectively. And the naming is consistent with the original image and ROI, as well.

The folder “json” contains 631 original annotation files in JSON format, that record the XY coordinates. The naming method is consistent with the images, in the format “subject number_laterality_visit number. json”. The JSON file can be opened using Notebook and some label tool software.

The python file, “draw.py”, records the codes for contours drawing. Running the codes in the “draw.py” can annotate the segmentation in json files on ROI of CFPs, and the results will be output in the folder “Annotated Images”.

## Technical Validation

### Dataset characteristics

There are 1115 records of 263 eyes from 144 glaucoma patients in the GRAPE dataset. Each visit of the patient serves as a record, and each eye has 3–9 times of visits. The interval between adjacent visit is more than 5 months. The mean age is 42.49 years old. Both genders and eye laterality remain a certain balance. The majority of patients in this dataset are OAG, may due to the requirement of complete examination for diagnosis of OAG and its long follow-up time for management. The detailed data characteristics are shown in Table 2.

**Table 2:** The data characteristics of the GRAPE dataset.

Item	Value
The number of patients	144
The number of eyes	263
The number of records	1115
Average age (SD)	42.51 (15.41)
Male (%)	75 (52.08%)
Right eye (%)	130 (49.43%)
OAG (%)	139 (96.53%)
Baseline MD (dB) (SD)	−7.11 (5.71)
Total MD (dB) (SD)	−7.21 (5.71)
Average follow-up times (SD)	4.21 (1.31)
Average follow-up years (SD)	2.51 (1.01)
Average interval years (SD)	0.81 (0.41)
The number of progression - PLR2 (%)	40 (15.21%)
The number of progression - PLR3 (%)	14 (5.32%)
The number of progression - MD (%)	27 (10.27%)

**Fig. 4:**
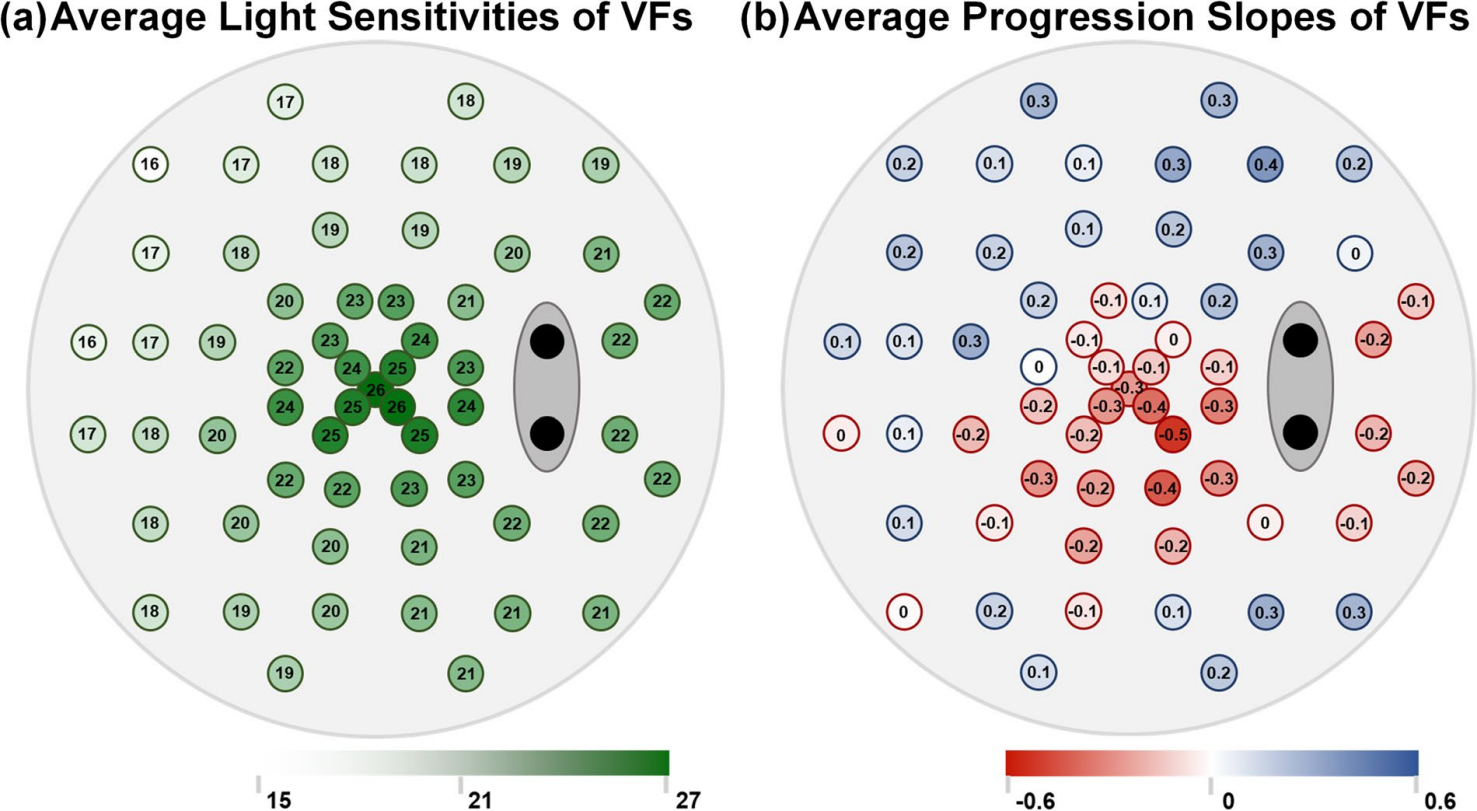
Statistics characteristics of visual field (VF) values of the GRAPE dataset. (**a**) Average light sensitivity values of each location of all follow-up VFs. (**b**) Average progression slopes of each location of VFs.

There are some statistic characteristics for VFs as well. Average light sensitivities of all follow-up VFs are shown in Fig. 4a. The original light sensitivities of central VF are highest and tend to be the last to be impaired. The nasal VF values are lowest due to the damage usually starting from the nasal side. Average progression slopes are shown in Fig. 4b. The negative progression is concentrated in the lower part of the paracentral field and temporal area. We suspect that some patients had been presented with impaired lateral VF, and the paracentral VFs were damaged subsequently.

### Baseline model validation for VF progression prediction

Because of the longitudinal and multi-modal data during the entire follow-up included, the GRAPE dataset is available for multi-purpose models training. It is important for patients that could be aware of their future condition at the first visit and it could also guide management. The prediction of whether VF progression or not has been realized in previous research from CFPs^11^ based on deep convolutional neural networks (CNNs). However, more experiments are needed to valid the prediction. In this part, we applied a common baseline model, Resnet-50, to show the technical validation of our dataset.

The CFPs of each eye at the baseline was considered as input, while the progression was defined by PLR2, PLR3 and MD slope as output, that each eye was classified as “progression” or “non-progression”.

The 263 CFPs were resized to 224 × 224 × 3 and the mean variance standardization method was used for normalization. We trained the basic CNN, Resnet-50, using cross entropy loss function for 264 epochs with a batch size of 4, with Adam optimizer. In addition, we used annealed cosine to update the learning rate from 10^−4^ to 10^−5^. The Receiver Operating Characteristic Curves were used to evaluate the performance of ResNet-50.

**Fig. 5:**
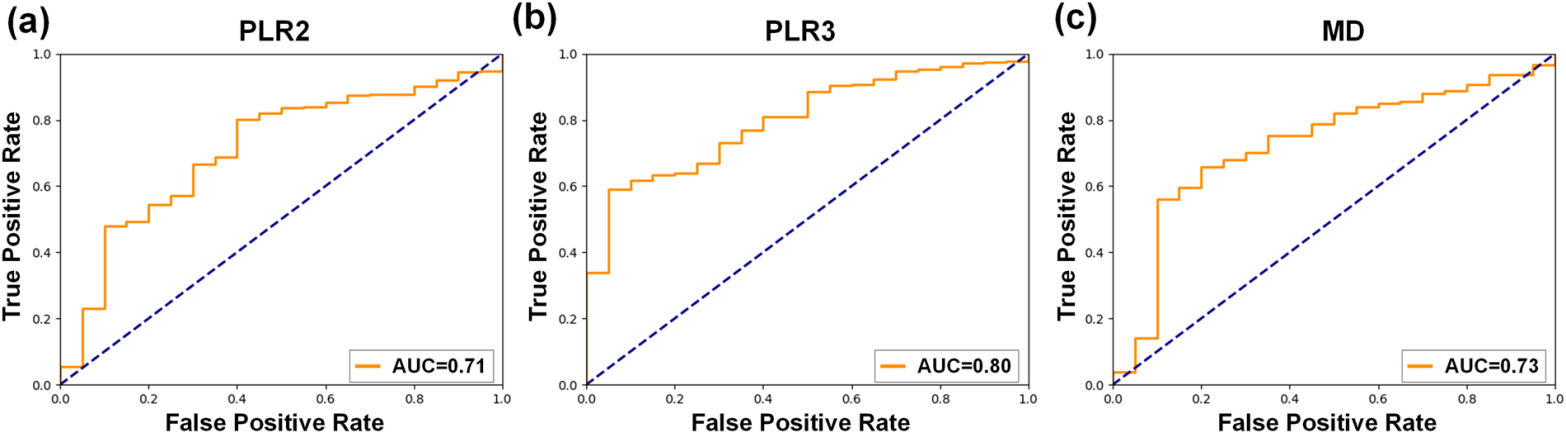
The Receiver Operating Characteristic Curves (ROC) of visual field (VF) progression prediction classification model on the GRAPE dataset. (**a**) The ROC of VF defined by PLR2. (**b**) The ROC of VF defined by PLR3. (**c**) The ROC of VF defined by MD.

We evaluated the results of predicting performance by the area under receiver operating characteristic curve (AUC), shown in Fig. 5. The AUC of the models of PLR2, PLR3 and MD is 0.71, 0.80 and 0.73. Besides, the accuracy of the models of PLR2, PLR3 and MD is 0.75, 0.91 and 0.81, respectively. It demonstrated that the GRAPE dataset could be used for VF progression prediction and the model with data partitioning defined by PLR3 has the best classification performance.

### Baseline model validation for VF estimation

SAP requires the effective cooperation and reliability of the patients with a relatively lengthy test time, and the results are subjective that affected by the test-retest variability^13–15^. Structural images or parameters have been used to estimate VF values in previous studies by DL models^16–19^. These studies explore the relationship between structure damage and function loss as well. The CFPs and VFs in our database can be utilized in this kind of VF estimation model. A baseline model based on ResNet-50 is proposed for example as below.

In this DL model, the CFPs, ROI of CFPs, ROI of CFPs with OD/OC segmentation of each eye at baseline were considered as input, while the values of VFs at each location as output. The input images were resized to 224 × 224 × 3.

We trained the same CNN, ResNet-50, with 50 epochs and a batch size of 16. The mean squared error was used as the loss function. The L2 regularization of 10^−5^ was imposed with Adam optimizer, and annealed cosine was used to update the learning rate from 10^−4^ to 10^−5^. Gradient clipping was carried out to restrict the ${\ell }_{2}$-norm of the gradient to 1.0. The mean absolute error (MAE), root mean square error (RMSE), and R-squared (R^2^), for measuring the difference between values predicted by the model and ground truth values, were used as metrics to evaluate the model estimating ability.

**Fig. 6:**
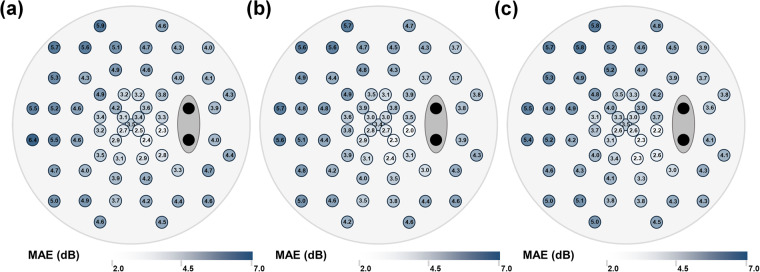
The mean absolute Error (MAE) of predicted visual field (VF) at each point. (**a**) The MAE of VF estimated from color fundus photographs (CFPs). (**b**) The MAE of VF estimated from region of interest (ROI) of CFPs. (**c**) The MAE of VF estimated from ROI of CFPs with optic disc and optic cup segmentation.

The MAE between predicted values and true values of each point are shown in Fig. 6, and the MAE of VF estimation from CFPs, ROI of CFPs, and ROI with OD/OC segmentation are shown in Fig. 6a–c, respectively. The prediction error is minimum at the lower part of the paracentral field, and maximum at the nasal field. The MAE, RMSE and R^2^ are listed in Table 3. The MAE of the models of CFPs, ROI of CFPs, and ROI with OD/OC segmentation is 4.143, 4.029, 4.107 respectively. It indicates that our model has the best predictive ability with ROI of CFPs, possibly because the features on ONH could be recognized by the model.

**Table 3:** The VF estimation error for baseline model on the GRAPE dataset.

	RMSE	MAE	R^2^
CFPs	5.627	4.143	0.263
ROI of CFPs	5.475	4.029	0.306
ROI with OD/OC segmentation	5.596	4.107	0.272

### Limitations and discussion

There are some limitations of the GRAPE dataset.

First, the bias of data selection is a shortcoming of this dataset. Only patients with OAG or ACG were included (mostly OAG), and the relatively low MD (−7.21 dB) meant more mild VF defects (MD ≥ −6 dB, stipulated by HPA criteria)^37^ patients were selected. Although some patients with moderate (−12 dB ≤ MD ≤ −6 dB) and severe (MD ≤ −12 dB) VF loss were excluded due to the VF measurement strategy and glaucoma surgery. This tendentious data inclusion would limit the generalizability of algorithm development. The performance of AI networks trained by this dataset may be affected in predicting the progression of other types of glaucoma or severe VF loss. We will consider collecting more severe VF data in the future.

Second, the inclusion data of 263 eyes with 1115 follow-up records is relatively small number for the dataset. The low number of patients was mainly owing to the setting of patient privacy, the selection of glaucoma types, the integrality of data, and the exclusion of combing with other serious eye diseases. It resulted in a small number of progressors defined by PLR3 and the possibility of overfitting of models. In the actual AI model development, the data augmentation could be a way to ameliorate the performance impact of this flaw.

Third, the detailed IOP control medications information, such as particular drug name and dosage, was not listed in this dataset. The EHR system had been updated for many times and other hospitals may dispense IOP control medicines during long the follow-up time span, leading to the information of medicines missing. To minimize the impact of treatment methods during the follow-up, we excluded the patients who had undergone glaucoma surgery to ensure that they were essentially medically-controlled.

In general, the GRAPE dataset could be used for prognostic prediction in glaucoma management and VF estimation for structure-function relationship exploration, that could advance the development of computer aided telemedicine in glaucoma.

## Usage Notes

The GRAPE dataset described in this article can be downloaded through the link mentioned before. Our dataset consists of multi-modal clinical information at the baseline and longitudinal VFs, CFPs and IOP during the follow-up of glaucoma patients. The GRAPE dataset is recommended to be used to develop AI models in prognosis evaluation and VF estimation. Besides, other prediction models, such as traditional ML models, could also be applied on this dataset. Users should properly cite this article and acknowledge the contributions in their study.

## Data Availability

The GRAPE dataset can be downloaded at the Figshare as mentioned above^36^. The codes of drawing annotations on ROI images are saved as the python file “draw.py”. The DL models applied in “Technical Validation” are not the as a part of the GRAPE dataset. We uploaded these models at the Figshare as two separated parts, “Baseline model validation for VF estimation” and “Baseline model validation for VF progression prediction”, that correspond to the 2 chapters. The parameters tuning is detailed in the article.
